# Variation in the sacroiliac joint in Felidae

**DOI:** 10.7717/peerj.11116

**Published:** 2021-05-11

**Authors:** Jean-Pierre Pallandre, Franck Lavenne, Eric Pellé, Grégory Breton, Mélina Ribaud, Vincent Bels

**Affiliations:** 1 Institut de Systématique Evolution Biodiversité (ISYEB-UMR7205, CNRS/MNHN/EPHE/UA), Sorbonne Université, Muséum national d’Histoire naturelle, Paris, France; 2 CNRS, INSB, Centre d’Etude et de Recherche Multimodale Et Pluridisciplinaire en imagerie du vivant, Bron, France; 3 Direction Générale des collections, Sorbonne Université, Museum national d’Histoire naturelle, Paris, France; 4 Panthera, New York, United States of America; 5 BioSP, INRAE, Avignon, France

**Keywords:** Felidae, Ilium, Evolution, Pelvis, Sacroiliac junction, Predatory behavior, Locomotion

## Abstract

Felidae species show a great diversity in their diet, foraging and hunting strategies, from small to large prey. Whether they belong to solitary or group hunters, the behavior of cats to subdue resisting small or large prey presents crucial differences. It is assumed that pack hunting reduces the per capita risk of each individual. We hypothesize that the sacroiliac articulation plays a key role in stabilizing the predator while subduing and killing prey. Using CT-scan from 59 felid coxal bones, we calculated the angle between both iliac articular surfaces. Correlation of this inter-iliac angle with body size was calculated and ecological stressors were evaluated on inter-iliac angle. Body size significantly influences inter-iliac angle with small cats having a wider angle than big cats. Arboreal species have a significantly larger angle compared to cursorial felids with the smallest value, and to scansorial and terrestrial species with intermediate angles. Felids hunting large prey have a smaller angle than felids hunting small and mixed prey. Within the *Panthera* lineage, pack hunters (lions) have a larger angle than all other species using solitary hunting strategy. According to the inter-iliac angle, two main groups of felids are determined: (i) predators with an angle of around 40° include small cats (i.e., *Felis silvestris, Leopardus wiedii, Leptailurus serval, Lynx Canadensis, L. rufus*; median = 43.45°), the only pack-hunting species (i.e., *Panthera leo*; median = 37.90°), and arboreal cats (i.e., *L. wiedii, Neofelis nebulosa*; median = 49.05°), (ii) predators with an angle of around 30° include solitary-hunting big cats (i.e., *Acinonyx jubatus, P. onca, P. pardus, P. tigris, P. uncia*; median = 31.80°). We suggest different pressures of selection to interpret these results. The tightening of the iliac wings around the sacrum probably enhances big cats’ ability for high speed and large prey control. In contrast, pack hunting in lions reduced the selective pressure for large prey.

## Introduction

In terrestrial Tetrapods, limb evolution is primarily linked to the transmission of propulsive forces from the limb to the body determining locomotion. The sacroiliac joint, firmly connecting the pelvic girdle to the vertebral column, is the center of transmission of these forces to the spine (Jenkins, 1971; Jenkins & Camazine, 1977; Barone, 1986; Polly, 2007; Kardong, 2015). Joint mobility is very limited, granting only some flexibility to the very solid union of the pelvis to the vertebrae (Barone, 1986; Abitbol, 1987; Kapandji, 2013). In mammals, the forward thrust of the hind limbs is brought into alignment with the travel vector and transferred to the vertebral column whenever this line of travel is horizontal or vertical (i.e., climbing; Taylor, 1989; Kardong, 2015). In the meantime, with increased speed, the long axis of the pelvis gets more aligned with the long axis of the sacrum (Romer, 1950; Taylor, 1989; Kardong, 2015). Within carnivorans, felids show a wide range of locomotor behaviors. They use their claws to hang on to substrate and all felids are excellent climbers (Gonyea, 1978). Some of them are able to forage in trees (e.g., *Leopardus wiedii, Neofelis nebulosa*). Even big *Panthera* species, which are considered as more terrestrial (e.g., *Panthera leo, P. tigris)*, or specialist runners (i.e., *Acinonyx jubatus*) can easily climb rocks or trees to find shelter (Meachen-Samuels & Van Valkenburgh, 2009a; Samuels, Meachen & Sakai, 2013; Martín-Serra, Figueirido & Palmqvist, 2014; Randau et al., 2016). All felids are also sprinters able to reach high speed with fast accelerations (Garland & Janis, 1993; Williams et al., 2014), either pursuing (including pounce-pursuit) or ambushing their prey (Eaton, 1970; Valkenburgh & Ruff, 1987; Scheel & Packer, 1991; Jenny & Zuberbühler, 2005; De Oliveira Calleia, Rohe & Gordo, 2009; Stanton, Sullivan & Fazio, 2015).

Limbs are involved in various actions such as playing, fighting, mating, territorial marking, and feeding, and this can impact the whole skeletal system. During prey capture, limbs are used in different ways to catch, take down and kill prey. The retractile claws of the forelimbs enable cats to grab their prey, and the benefits of forearm pronation-supination during prey manipulation have been well described (Gonyea & Ashworth, 1975; Gonyea, 1978; Russell & Bryant, 2001; Meachen-Samuels & Van Valkenburgh, 2009a; Stanton, Sullivan & Fazio, 2015; Cuff et al., 2016a; Viranta et al., 2016). However, the role of hind limbs in relationship with predation remains poorly investigated (Samuels, Meachen & Sakai, 2013; Cuff et al., 2016b).

Felidae show a remarkable unicity in morphology (Cuff et al., 2015; Gittleman, 1985; Parés-Casanova & De la Cruz, 2017; Werdelin & Wesley-Hunt, 2014). There are, however, major morphological variations when it comes to mass and size, with cats ranging from less than 1 kg to more than 300 kg (Cuff et al., 2015). With a body mass above 14.5–21.0 kg, carnivores increase their energy intake net gain rate by adopting a large prey feeding strategy (Carbone, Teacher & Rowcliffe, 2007). Although felids prey selection and energy intake still remains discussed (Carbone et al., 1999; Carbone, Teacher & Rowcliffe, 2007; Carbone, Pettorelli & Stephens, 2010; Cuff et al., 2015, 2016a; Tucker, Ord & Rogers, 2016), with increasing body mass, felids tend to select a wide range of prey size switching from small to mixed and big prey (Radloff & Du Toit, 2004; Meachen-Samuels & Van Valkenburgh, 2009a,b; Sicuro & Oliveira, 2011; Hartstone-Rose, Perry & Morrow, 2012; Selvan et al., 2013; Cuff et al., 2015, 2016a).

This diversity in diet is supported by variation in foraging, hunting and killing strategies (Wang et al., 2004; Gittleman, 2013). For example, small cats (e.g., Domestic cat, Bay cat, Caracal and Ocelot lineages species) tend to play with their prey and end up killing it quickly with a bite to the spine or head. In contrast, a suffocating throat bite is known to be the usual lethal bite in big cats (e.g., *Panthera* sp.; Kitchener et al., 2010). However, some *Panthera* species can switch from typical throat bite to muzzle bite (e.g., *P. leo)*, or at the back of the head (e.g., *P. onca;* Schaller & Vasconcelos, 1978; Palmeira et al., 2008; Kitchener et al., 2010). Although individuals of solitary species catch large prey on their own (Kleiman & Eisenberg, 1973; Macdonald, 1983; Grzimek, 1990; Bailey, Myatt & Wilson, 2013; Gittleman, 2013) some cooperation can occasionally occur (e.g., male cheetahs) by forming stable coalitions (Caro, 1994; Radloff & Du Toit, 2004). Asian and African lions (*P. leo*) are the only extant felids living in family groups that use a pack-hunting strategy, increasing their chances of killing very large prey (Kleiman & Eisenberg, 1973; Sunquist & Sunquist, 1989; Rudnai, 2012; Bailey, Myatt & Wilson, 2013; Mukherjee & Heithaus, 2013). During prey capture, predators must avoid prey weapons (e.g., horns, antlers, hooves; MacNulty, Mech & Smith, 2007; Mukherjee & Heithaus, 2013). Direction and position of the attack coupled with predator behavior are main components to avoid injuries from prey. Faced with large prey, predator’s behavior is different depending on whether it hunts alone or in a group (Kleiman & Eisenberg, 1973; Macdonald, 1983; Bailey, Myatt & Wilson, 2013; Gittleman, 2013). Also, the way groups of African lions live and hunt minimizes the risk for each individual (Scheel & Packer, 1991; Scheel, 1993; Sheldon, 2013).

**Figure 1:**
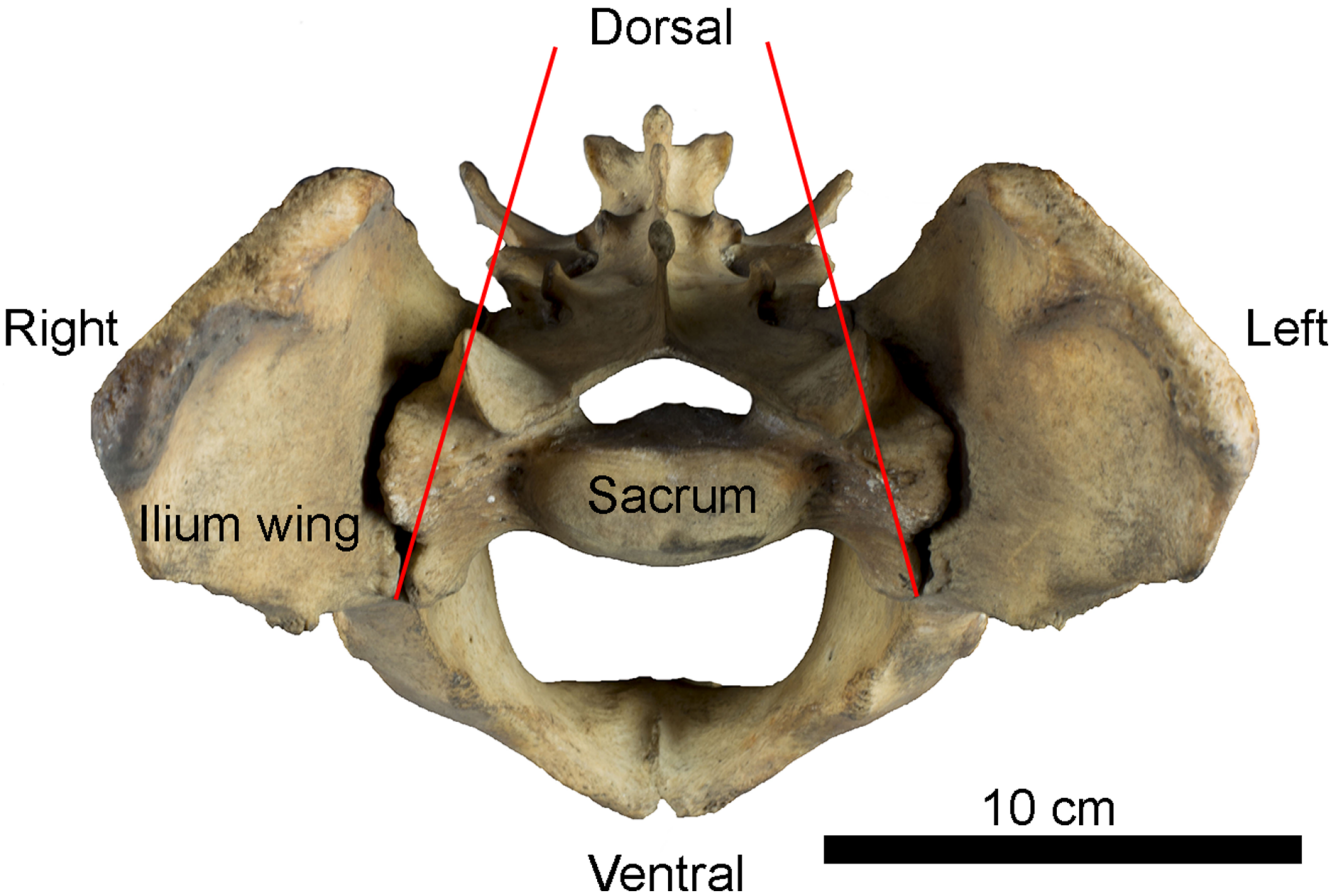
Example of sacro-iliac joint in *Panthera tigris* (Felidae). Anterior view of the pelvis showing the position of iliac wings around the sacrum. Red lines indicate inter-iliac angle.

Recent studies in carnivorans revealed that the pelvis shape was influenced by phylogeny and body mass but not locomotor behavior (Lewton et al., 2020). Furthermore, investigation of ecological correlates to the variation of the sacro-iliac joint in felids demonstrated that body mass, prey selection, and bite type impacted the auricular surface, where no effect of locomotor specialization was found (Pallandre et al., 2020). In our paper, we hypothesize that the angle between both sacroiliac joints in the transverse plane (i.e., inter-iliac angle; Fig. 1), plays a key role in the sacroiliac interlocking performance, and we tested the effect of locomotion and predation on this angle. We assess that the tightening of the iliac wings around the sacral wings enhances joint stiffness and transmission of forces from the hind limbs to the spine, because forces resulting from pulling on food in addition to bite force may also contribute to feeding success as demonstrated in various carnivorous species (Helfman & Clark, 1986; Fish et al., 2007; D’Amore et al., 2011; Pallandre et al., 2020). We assume that an acute angle provides a functional advantage in this transmission when predators capture and struggle with large prey. Therefore, by using CT-scan slides, we compared the inter-iliac angle in representative Felidae species showing different locomotor behaviors and exploiting small and large prey. Finally, functional analysis of the predatory behavior of studied species provides useful data to help discuss the morphological properties of the inter-iliac angle.

## Materials and Methods

### Data

The data set for this study included 59 adult individuals coxal bones from 12 species of extant Felidae (Table 1). Sample specimens were selected for ossification stage of the pubic symphysis and included males and females (see Supplemental Information). All specimens were obtained from the collection “Mammifères et Oiseaux” of the Museum National d’Histoire Naturelle (Paris, France) and are available upon request through the Museum service Collhelper. No permits were required for the described study.

**Table 1:** Morphological and behavioral characteristics of felids and species used to compare the inter-iliac angle.

Species	Number of specimens	Locomotion classes^(1)^	Prey size preference^(2)^	Hunting strategy^(3)^	Average body weight (kg)^(4)^	Body mass range (kg)^(2)^
*Acinonyx jubatus*	7	Cursorial	Large	Solitary	53.5	40–65
*Felis silvestris*	2	Scansorial	Small	Solitary	5.5	3–6
*Leopardus wiedii*	1	Arboreal	Small	Solitary	3.3	2–4
*Leptailurus serval*	1	Terrestrial	Small	Solitary	13.4	8–18
*Lynx canadensis*	2	Terrestrial	Mixed	Solitary	11.2	5–17
*Lynx rufus*	2	Scansorial	Mixed	Solitary	11.2	4–16
*Neofelis nebulosa*	1	Arboreal	Mixed	Solitary	19.5	11–25
*Panthera leo*	13	Terrestrial	Large	Pack	185.0	110–250
*Panthera onca*	5	Scansorial	Large	Solitary	105.7	36–120
*Panthera pardus*	13	Scansorial	Large	Solitary	59.0	28–65
*Panthera tigris*	10	Terrestrial	Large	Solitary	185.5	75–325
*Panthera uncia*	2	Scansorial	Large	Solitary	50.0	22–52

**Notes:**

(1) Samuels, Meachen & Sakai (2013).

(2) Meachen-Samuels & Van Valkenburgh (2009a).

(3) Sunquist & Sunquist (2017).

(4) Sicuro & Oliveira (2011).

### Measurement of the inter-iliac angle

**Figure 2:**
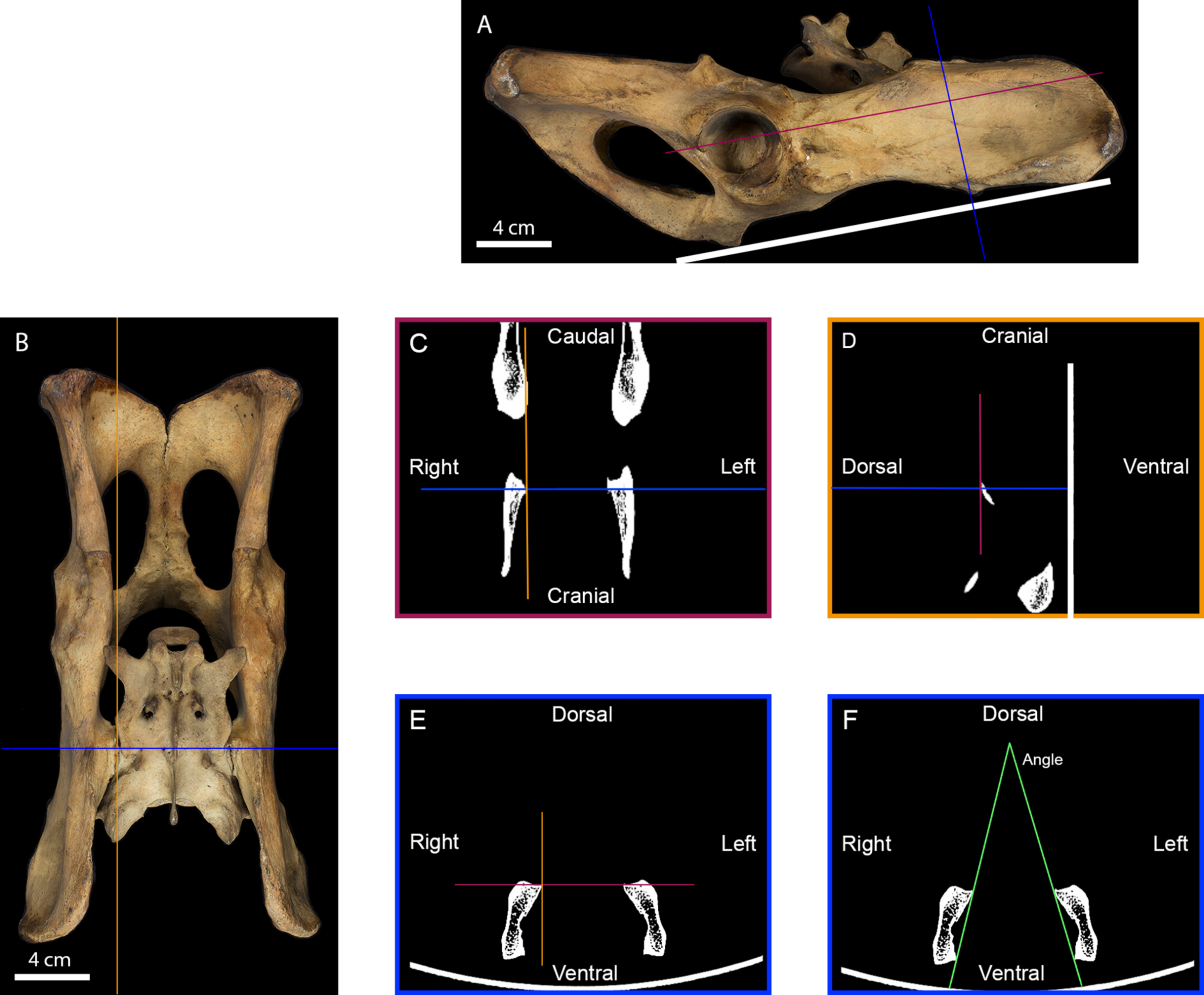
Measurement of the inter-iliac angle. (A) Right lateral view of a tiger pelvis showing the position on the scanner bed (white line), the transverse (blue line) and the frontal (purple line) planes used to select the scanner cross-section. (B) Dorsal view of the pelvis showing the transverse (blue line) and parasagittal (orange line) planes used to select the scanner slides. (C) Scanner frontal slide of the coxal bone showing the transverse and parasagittal planes meeting the medial-most point of the right iliac articular surface. (D) Scanner parasagittal slide of the coxal bone showing the transverse and frontal planes meeting the dorsal-most point of the right iliac articular surface. (E) Scanner transverse slide of the coxal bone going through the medial-most and dorsal-most point of the articular surface showing the frontal and parasagittal planes meeting at that point. (F) Selected scanner transverse slide showing the lines going from the dorsal-most to the ventral-most point of each articular surface. The angle between these lines was calculated in degrees to evaluate the angle between the right and the left iliac articular surfaces.

The orientation of the sacroiliac joints relative to the median plane was quantified measuring the tightening of the iliac wings around articular surfaces of the sacrum. All specimens were scanned at the CERMEP Imagerie du vivant (Bron, France) with a Siemens Healthcare mCT/S 64. The angle between both iliac auricular surfaces (°) was calculated on the transverse slide of ilium bones scanner images (Fig. 2). The coxal bones were positioned ventrally lying on the CT-scan bed, with the median plane of the scanner meeting the median plane of the bones going through the pubic symphysis. Thus, cross-section of obtained images corresponded to cross-section of the bone. The iliac auricular surface of the right ilium was selected and the cross-section going through the dorsal-most and medial-most point of the auricular surface was chosen on a 3D Multiplan reconstitution by using OsiriX software. On this slide, the line going from the dorsal-most point to the ventral-most point of the articular surface on each side of the bone was selected. The inter-iliac angle between the right and the left lines was calculated in degrees by using OsiriX software and was used for statistical analyzes. We evaluated pelvis size by calculating the distance between the lateral-most points (cm) of each ischial tuberosity (DIT) on a CT-scan cross section of coxal bones going through these points by using OsiriX software (see Supplemental Information). Repeating each procedure 10 times on the same sample with less than 5% variation in the measured values validated the protocol.

### Statistical analyzes

Because all of the recorded data did not follow the assumptions of normal distribution and homoscedasticity, we used non-parametric statistical tests (Sokal & Rohlf, 1981). Descriptive analyzes (median, first and third quartiles) were performed on the set of data. The level of significance for all tests was *p*-value < 0.05, and Bonferroni correction was calculated for all non-parametric tests used in this paper (Kruskal–Wallis *K*-test; Dunn post-hoc tests and Mann–Witney *U*-test) as previously suggested (Everitt & Dunn, 2001).

### Effect of body mass

Because the body mass is related to the skeletal size (Cuff et al., 2015), and pelvis size correlates with body size (Martín-Serra, Figueirido & Palmqvist, 2014; Lewton, 2015; Lewton et al., 2020), DIT was used to estimate the effect of body mass on the inter-iliac angle. All data were Log transformed to calculate this relationship in the studied species.

### Effect of locomotor behavior

We classified felid species into four locomotor groups (Table 1) following the categories defined by Samuels, Meachen & Sakai (2013): (i) terrestrial, species that rarely swim or climb; (ii) scansorial, species capable of climbing but do not forage in trees; (iii) arboreal, species that climb and actively forage in trees; (iiii) cursorial, species that regularly display rapid locomotion. We used a Kruskal–Wallis *K*-test followed by Dunn post-hoc test (Dunn, 1964) to test the angle in locomotor classes suggested by these authors.

### Effect of predation

Beside the potential effect of locomotor behavior on the inter-iliac angle, we tested the effect of felid prey selection. Table 1 provides all factors and classes tested on the angle. First, we followed Meachen-Samuels & Van Valkenburgh (2009a) prey preference classes to test this effect on the angle by using a Kruskal–Wallis *K*-test followed by Dunn post-hoc test (Dunn, 1964). Second, since regular cooperative hunting is found in *Panthera* lineage only, we tested pack- *vs* solitary-hunting strategies in this lineage by using a Mann–Whitney *U*-test.

### Predatory behavior

**Figure 3:**
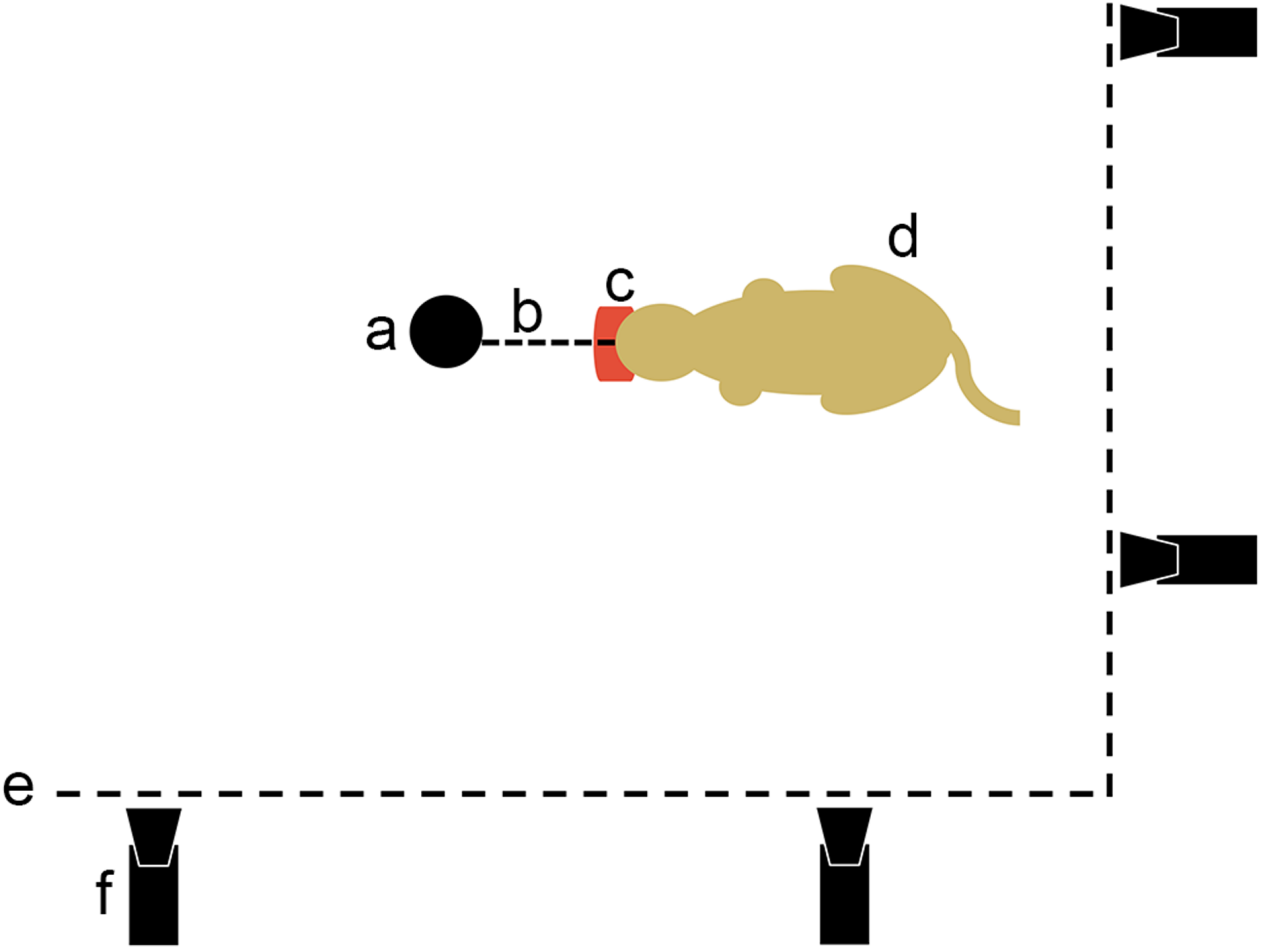
Movie protocol used to observe felids’ predatory behavior pulling on food. a, opportunistic fix point in the pen (e.g., tree); b, link between the fix point and the food; c, piece of meat; d, cat pulling on its food; e, pen fence; f, camera.

We filmed nine species of felids (i.e., *A. jubatus, L. wiedii, Lynx Canadensis, L. rufus, P. leo, P. onca, P. pardus, P. tigris, P. uncia*) feeding on a piece of meat fixed in place to demonstrate felids ability to produce a recoil force through their hind limbs. As part of enrichment process when feeding the cats, the food was tied to a strong fixed point in a corner of the pen. Cameras were mounted on both sides of the corner of the pen to get side views of felids pulling on resisting food (Fig. 3). All data were obtained in the context of a legal agreement (SJ MNHN 289-15) between the Muséum national d’Histoire naturelle de Paris, France (YSIEB-UMR7205 V. Bels) and Le Parc des Félins, Domaine de la Fortelle, RD402, 77540 Lumigny, France (P. Jardin). This agreement was approved by the legal department of the Muséum national d’Histoire naturelle and did not include any animal manipulation. No permits were required for the described study.

## Results

### Body mass

**Figure 4:**
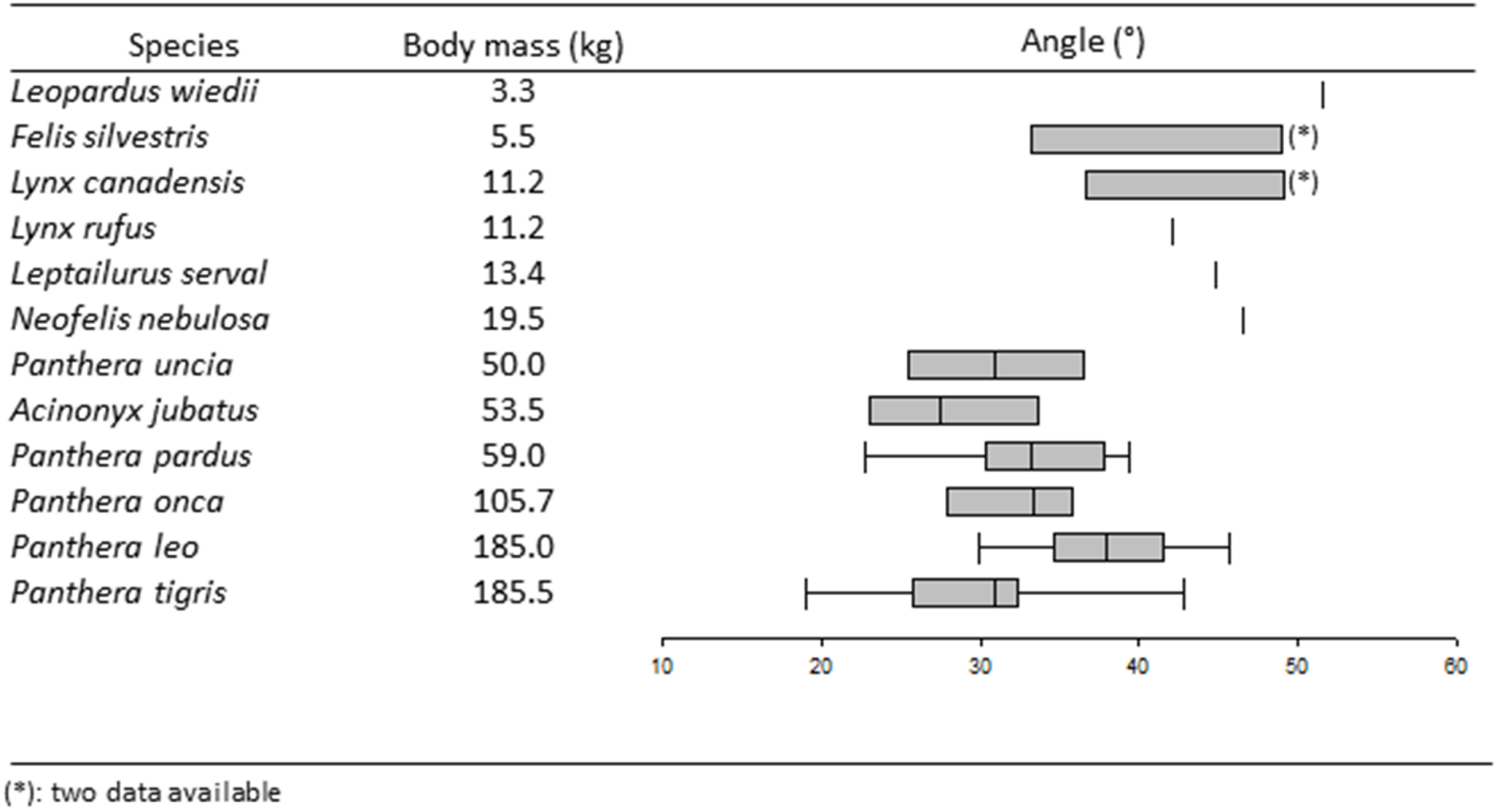
Boxplots and whiskers showing the statistical distribution of measured inter-iliac angle in studied species.

Figure 4 and Table 2 show all the descriptive analyzes on the inter-iliac angle used in this study. A Log-Log linear regression between the inter-iliac angle and DIT shows that the angle decreases with increasing body mass (R^2^ = 0.28; ddl = 1, 55; F = 29.07; *p* < 0.0001).

**Table 2:** Descriptive statistics of the inter-iliac angle (°) measured in this study.

Factors	Median	Q_1_	Q_3_
Locomotion			
Cursorial	27.50	24.85	32.10
Terrestrial	36.10	30.72	40.35
Scansorial	33.85	30.45	37.52
Arboreal	49.05	47.77	50.32
Prey preference			
Small	46.85	41.90	49.57
Mixed	42.10	36.60	46.50
Large	31.40	27.60	35.20
Hunting Strategy in *Panthera*			
Solitary	31.80	29.05	36.40
Pack	37.90	35.70	40.60

**Note:**

Q1 first quartile; Q3 third quartile.

### Locomotor behavior

The locomotion mode has a significant effect on the angle (K = 11.13, *p* < 0.05). The Dunn post-hoc test separates cursorial felids (*A. jubatus*, median = 27.5*°*) and arboreal felids (*N. nebulosa and L. wiedii*, median = 49.05°) into different groups. Scansorial and terrestrial felids belong to a same group.

### Effect of predation

A significant effect on the angle is shown through prey preference, by using he Kruskal–Wallis test (K = 16.14, *p* < 0.05). The Dunn post-hoc test separates felids hunting on large prey (median = 31.4°) from felids hunting on mixed and small prey. Within the *Panthera* lineage, the *U*-test shows that *P. leo*, the only species using pack-hunting strategy, has an angle significantly wider (median = 37.90°) than in all other species showing solitary-hunting strategy (median = 31.80°).

### Predatory behavior

**Figure 5:**
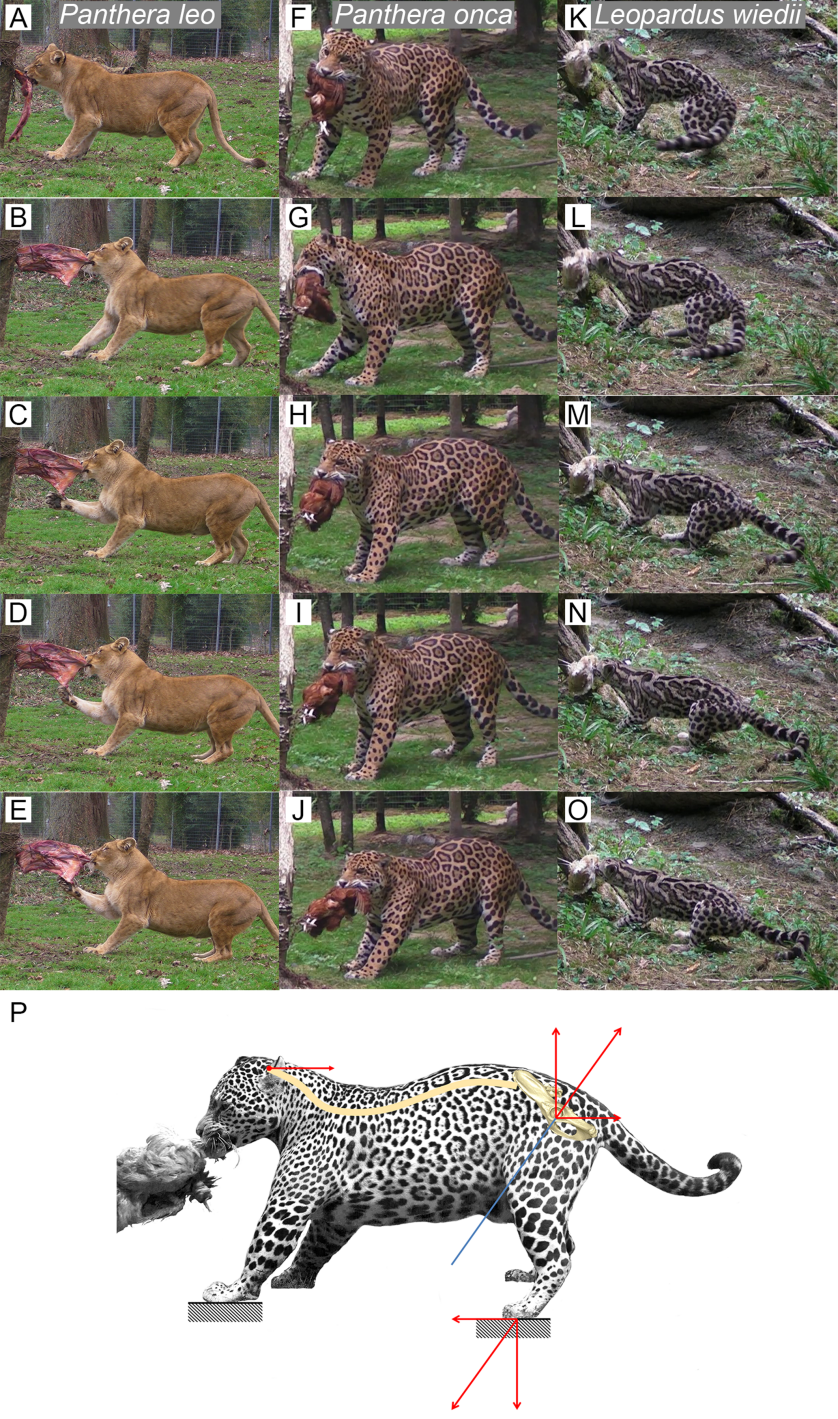
Functional hypothesis of forces transmission through hind limbs when felids pull on food. (A–O) Personal observations of felid behavior when coping with food they cannot carry away: (A–E) *Panthera leo*, (F–J) *Panthera onca*. (K–O) *Leopardus weidii*. (A, F, K) Biting. (B–C, G–H, L–M) Pulling the food by stepping backwards. (D, I, N) Adjusting hind limbs. (E, J, O) Final pulling without stepping, increased contact on the ground with the whole paw (plantigrade position), extension of the back. Front limbs can either push on the ground or manipulate the food when the cat pulls it backwards. (P) Biomechanical model of hind limb forces transmission produced when predator fight with a resisting prey. Blue line: femur long axis. Red arrows: force vectors produced through the hind limb when pushing on the ground and transmission from the sacro-iliac joint to the head through the spine.

We observed several felids behavior interacting with food they could not remove (Fig. 5). Without copying the behavior of predators when struggling with prey, this protocol shows an ability of felids to produce a recoiling force with the hind limbs. Once the bite grip is performed, felids pull on the food by stepping backwards. Front and hind limbs push the body backwards while the jaw maintains the bite. Then, they adjust the hind limbs symmetrically and flex their lumbar column and their hind limbs underneath the body. They increase contact to the ground by putting down the whole paw (plantigrade position). From that position, without stepping, they perform a final pull with their hind limbs and back extension. During the recoil motion when cats pull the food backwards, fore limb can either push on the ground or manipulate the food.

## Discussion

### Body mass

Our results show that body mass estimated by the skeletal size (Cuff et al., 2015) significantly explains the inter-iliac angle as shown by Log-Log linear regression. But less than 30% of the angle variability (R^2^ = 0.28) is explained by this allometric relationship. Ecological and associated behavioral traits (e.g., habitat, locomotor specialization and predatory behaviors) remain major stressors influencing the inter-iliac angle variability in Felidae. Here we discuss two main traits that impact the inter-iliac angle variability in these predators: (i) locomotion and (ii) predation.

### Locomotor behavior

The Kruskal–Wallis *K*-test shows that locomotion has a significant effect on the inter-iliac angle. *A. jubatus*, with the smallest angle (median = 27.50°), is separated from all other felids. In addition to being the only feline cursorial representative, the cheetah is also the greatest sprinter with a maximum speed of around 110 km/h (Taylor et al., 1974; Garland & Janis, 1993; Hudson et al., 2011; Hudson, Corr & Wilson, 2012), almost double the maximum speed of all other cursorial carnivorans (Garland & Janis, 1993). Sprint specialization in cheetahs correlates with long and strong hind limb bones with a long and narrow pelvis (Hopwood, 1947; Taylor et al., 1974; Buss & Duntley, 2008; Hudson et al., 2011). Even though cheetahs have more muscle mass in the hind limb than other high-speed mammals, they have less muscle mass around the hip compared to, for instance, the greyhound, with a maximum speed of 61 km/h (Hudson et al., 2011). To obtain the power required for acceleration through hip extension, Hudson et al. (2011) suggest that the substantial amount of back musculature in cheetah provides additional power. During hip extension, the contraction of spinal erectors (i.e., m. *Erector spinae*), with medial insertion on the iliac wings (Barone, 1986), tightens the iliac wings around the sacrum. This tightening combined with the W-inner shape of iliac auricular surface described in big cats (Pallandre et al., 2020) probably enhances the locking ability of the sacroiliac junction. A smaller angle increases the locking effect of iliac wings tightening and could bring a benefit for pelvis stabilization at the connection between hind limbs and the vertebral column, and resist pitching moments that might occur during accelerations and high speed gallop.

The Kruskal–Wallis *K*-test also separates arboreal felids (i.e., *N. nebulosa* and *L. Wiedii*,) from all other felids. This test groups together two species with the largest angles (median = 49.05°) belonging to different lineages: *Leopardus cat* lineage (*L. wiedii)* and *Panthera* lineage (*N. nebulosa*) (Nyakatura & Bininda-Emonds, 2012). The margay (*L. Wiedii)* is a small species (2.3–4.9 kg; Table 1) able to forage small prey in trees. Margays are capable of climbing down trees headfirst, thanks to their ability to rotate their tarsal joint 180° (De Oliveira, 1998; De Oliveira Calleia, Rohe & Gordo, 2009; De Bianchi et al., 2011; Morales et al., 2018). In contrast, the clouded leopard (*N. nebulosa)* is a big cat (12.0–23.0 kg; Table 1) able to hunt and catch prey much bigger than itself (Rabinowitz, Andau & Chai, 1987; Sicuro & Oliveira, 2011; Sunquist & Sunquist, 2017). In these species, with early phylogenetic separation (Nyakatura & Bininda-Emonds, 2012) showing different predatory behaviors, the influence of locomotor behavior could play a key role on the inter-iliac angle. A wide angle might favor the necessary abduction described for climbing (Martín-Serra, Figueirido & Palmqvist, 2014; Cuff et al., 2016b). The group including scansorial and terrestrial felids shows intermediate angle values (Table 2). Therefore, felid climbing frequency (that remains to be certified) does not impact the inter-iliac angle in these species.

### Predation

Several body mass thresholds, varying from 14.5 to 21.5–25 kg, were determined to explain carnivores prey selection in relationship with their energy intake (Carbone et al., 1999; Carbone, Teacher & Rowcliffe, 2007). More recently, a comparative analysis looking at cost of carnivory in terrestrial and marine mammals determined a close threshold of 11 kg (Tucker, Ord & Rogers, 2016). Furthermore two optimal weights of felids in relation to their diet have been determined: one around 5 kg and the other around 100 kg (Cuff et al., 2015). The difference in the inter-iliac angle measured between small cats preying on small or mixed prey and big cats preying on large prey confirms that the shape of the joint is significant for species whose optimum mass is around 5 kg and around 100 kg (Cuff et al., 2015). In big cats selecting large prey (median = 31.40°), the inter-iliac angle is around 10° to 15° more acute than in small cats preying on small (median = 46.85°) or mixed prey (median = 42.10).

**Figure 6:**
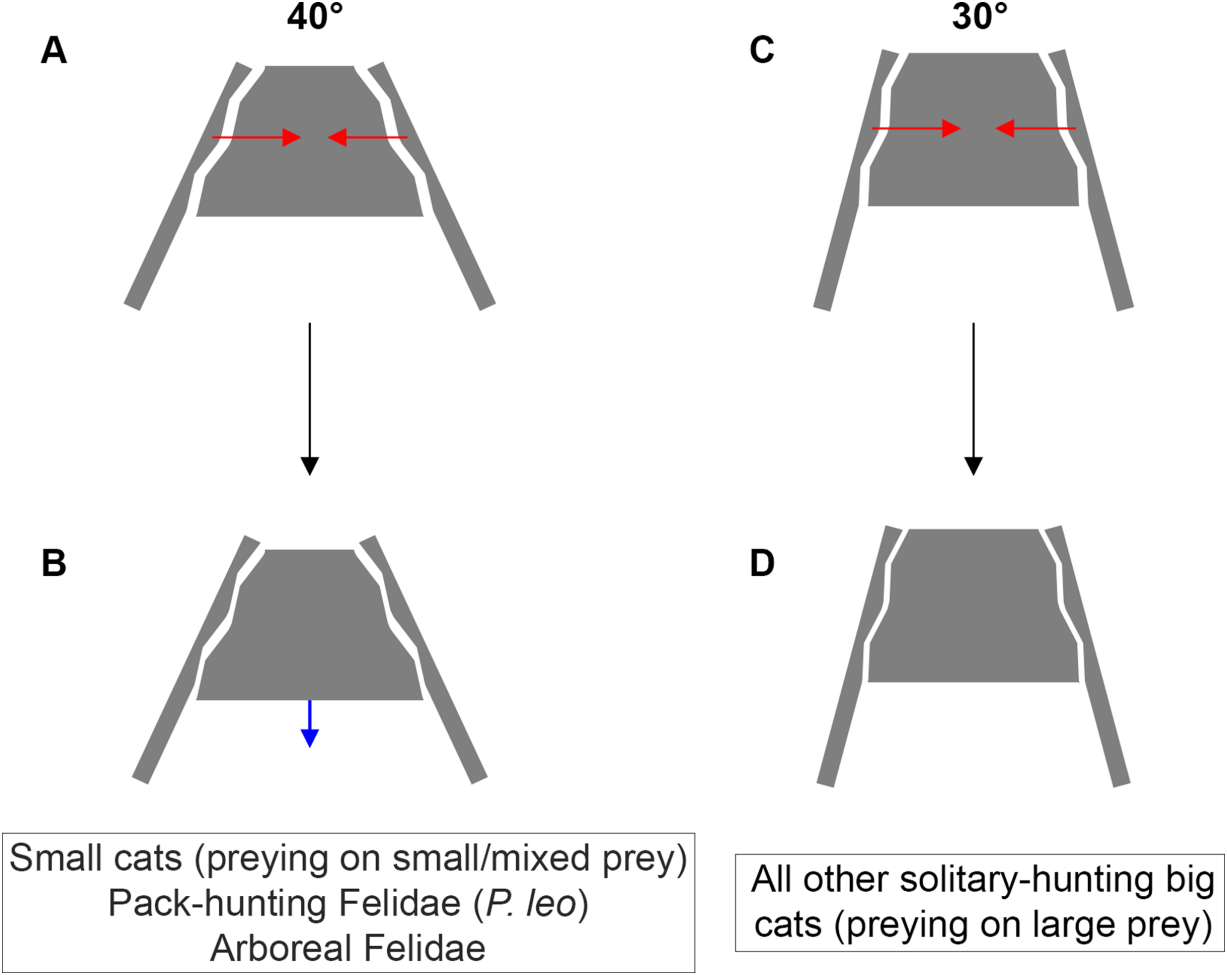
Inter-iliac angle in the interlocking process during predatory behavior in Felidae. (A–B) Predators with an angle of around 40° include solitary-hunting small cats, pack-hunting Felidae (lions) and arboreal Felidae. (C–D) Predators with an angle of around 30° include all solitary hunting big cats. The red arrows represent the forces produced through the post-cranial system and applied on the iliac wings (A, C). The blue arrow represents the possible slippage of the sacrum (B). We hypothesize that such movement is not possible for an inter-iliac angle of around 30° (D).

Functionally, we hypothesize that the tightening of the sacroiliac angle has a beneficial effect on predator’s ability to subdue large prey. In big cats, the lethal snap bite used for small prey switches to sustained bite for large prey (Schaller & Vasconcelos, 1978; Palmeira et al., 2008; Kitchener et al., 2010). Among all different forces involving the postcranial system during the struggle, producing a momentum opposing the prey escape is crucial to keep the bite grip. Pallandre et al. (2020) suggested that a full transmission of such momentum from hind limbs to the cranium could be favored by increased locking of the sacroiliac junction. Indeed, the tensile forces produced from the postcranial musculature have been described as part of the feeding behavior of several predators (Helfman & Clark, 1986; Fish et al., 2007; D’Amore et al., 2011; Pallandre et al., 2020). In this study, we observed the ability of felids to produce a recoiling force through fore and hind limb when coping with food that they cannot carry away (Fig. 5). This force seems to be produced by hind limb, in particular when front limb is handling the food. We suggest that such a recoil force stabilizes the prey and favors the sustained bite of the predator during the struggle. Once the bite grip is performed, felids flex their lumbar column and their hind limbs underneath the body. Then, hind limbs extend in a recoil movement and the lumbar column extends too. Due to the backward forces generated by the limbs, the pelvis moves backwards as well and *Erector spinae* muscles contract during the back extension. We assume that this muscle tension, due to medial insertions of the muscles on the iliac wings (Barone, 1986) tightens the iliac wings around the sacrum. In addition to the complex topography of the sacroiliac articular surface in big cats, preventing slippage, and to the elevated ridge observed in the *Panthera* lineage, preventing dorso-ventral gliding of the sacrum (Pallandre et al., 2020), an acute inter-iliac angle might provide better locking property of the sacroiliac junction during the recoil movement of the body (Fig. 6). We suggest that during the sustained bite, an acute inter-iliac angle prevents dorso-ventral slippage of the sacrum, reinforces whole-body stiffness and increases the transmission of forces from hind limb to the head (Figs. 5, 6).

All *Panthera* species show the highest prey-mass/predator-mass ratios ranging between 1.9 and 2.7 (Sicuro & Oliveira, 2011). Lions are the only felid species showing a regular pack-type hunting strategy (Scheel & Packer, 1991; Stander, 1992; Heinsohn & Packer, 1995). In *P. leo*, the inter-iliac angle is around 8° larger than in all solitary *Panthera* species (Table 2). Although some felids are opportunistic cooperative hunters (Kleiman & Eisenberg, 1973; Bailey, Myatt & Wilson, 2013; Gittleman, 2013), and lions are able to catch big prey solitarily (Sicuro & Oliveira, 2011), pack- *vs* solitary-hunting strategy has a significant effect on the angle. In *P. leo*, several individuals can simultaneously subdue the prey and the role of each individual is probably less constraining to fight against prey resistance. We hypothesize that the caudo-cranial momentum from hind limbs to the head during prey pulling is reduced for each pack-hunting individual. Furthermore, it is assumed that pack hunting reduces the per capita risk of each individual as demonstrated in wild African dogs (Scheel & Packer, 1991; Scheel, 1993; Sheldon, 2013). Therefore, we suggest a release of the effect of predatory behavior on the inter-iliac angle in felids using pack-hunting strategy to kill large prey.

## Conclusion

A trade-off between locomotion and predation, suggested as being one of the major evolutionary pressures on body mass, is recognized in carnivorans (Carbone et al., 1999; Carbone, Teacher & Rowcliffe, 2007; Meachen-Samuels & Van Valkenburgh, 2009b, a; Meachen, O’keefe & Sadleir, 2014; Cuff et al., 2015, 2016a; Pallandre et al., 2020). Functionally, predation depends on predator properties (e.g., cognition, nutrient and energy requirement, mass, behavior) and prey characteristics (e.g., density, behavior, ability to escape and defend, velocity, acceleration). According to our investigation, it is possible to relate the inter-iliac angle with: (i) body mass (the angle value decreases with increasing body mass) (ii) locomotor behavior and (iii) the predatory behavior repertoire. An acute angle could favor sacro-iliac junction stiffness and locking property (Fig. 6). This stiffness could bring a benefit for body support and body stabilization during high-speed locomotion and big prey subduing. Whereas forelimbs stabilize the prey-predator pair and can manipulate the prey during the bite (Gonyea & Ashworth, 1975; Gonyea, 1978; Stanton, Sullivan & Fazio, 2015; Viranta et al., 2016), hind limbs are involved in the transmission of forces to the vertebral column through the sacroiliac joint to subdue the prey. In conclusion, the inter-iliac angle is characterized by a morphological shift in relationship with locomotion and predatory behavior during the evolution of Felidae: (i) Felidae with an angle of around 40° include small cats, that hunt on small and mixed prey, the pack-hunting lion, and arboreal species, (ii) Felidae with an angle of around 30° include all other solitary-hunting big cats, that hunt on large prey (Fig. 6).

This study confirms former functional analysis on the biomechanical constraints of various behavioral and ecological factors on the sacroiliac joint morphological traits (Pallandre et al., 2020). Other ecological and behavioral stressors (e.g., hunting tactics, social behavior, growth conditions, habitat proximal characteristics, personality and individual live) should be tested on the set of data to provide an overview of the functional trade off between all activities performed by felids and the morphology of their pelvic girdle. This analysis could also be generalized to a larger set of data of carnivores with various hunting strategies (e.g., Canidae), and diet regime including derived herbivorous diet (e.g., *Ailuropoda* sp.). Finally, our study could provide the opportunity to discuss hunting strategies in fossil taxa on the basis of morphological traits of their pelvic girdle (e.g., *Panthera atrox* and *Smilodon* sp).

## Supplemental Information

10.7717/peerj.11116/supp-1 Supplemental Information 1 List of specimens and angle value.

## Additional Information and Declarations

### Field Study Permissions

The following information was supplied relating to field study approvals (i.e., approving body and any reference numbers): Movies on living animals were obtained in the context of a legal agreement (SJ MNHN 289-15) between the Muséum national d’Histoire naturelle de Paris, France (YSIEB-UMR7205 Vincent Bels) and Le Parc des Félins, Domaine de la Fortelle Lumigny, France (Patrick Jardin).

### Data Availability

The following information was supplied regarding data availability: The data is available at Morphosource: DOI 10.17602/M2/M167452. DOI 10.17602/M2/M167453. DOI 10.17602/M2/M167454. DOI 10.17602/M2/M167455. DOI 10.17602/M2/M167456. DOI 10.17602/M2/M167457. DOI 10.17602/M2/M167458. DOI 10.17602/M2/M167459. DOI 10.17602/M2/M167460. DOI 10.17602/M2/M167461. DOI 10.17602/M2/M167462. DOI 10.17602/M2/M167463. DOI 10.17602/M2/M167464. DOI 10.17602/M2/M167465. DOI 10.17602/M2/M167466. DOI 10.17602/M2/M167468. DOI 10.17602/M2/M167470. DOI 10.17602/M2/M167471. DOI 10.17602/M2/M167472. DOI 10.17602/M2/M167473. DOI 10.17602/M2/M167474. DOI 10.17602/M2/M167279. DOI 10.17602/M2/M167475. DOI 10.17602/M2/M167476. DOI 10.17602/M2/M167524. DOI 10.17602/M2/M167525. DOI 10.17602/M2/M167526. DOI 10.17602/M2/M167527. DOI 10.17602/M2/M167528. DOI 10.17602/M2/M167529. DOI 10.17602/M2/M167530. DOI 10.17602/M2/M167531. DOI 10.17602/M2/M167532. DOI 10.17602/M2/M167534. DOI 10.17602/M2/M167650. DOI 10.17602/M2/M167651. DOI 10.17602/M2/M167652. DOI 10.17602/M2/M167653. DOI 10.17602/M2/M167654. DOI 10.17602/M2/M167667. DOI 10.17602/M2/M167682. DOI 10.17602/M2/M167704. DOI 10.17602/M2/M167707. DOI 10.17602/M2/M167709. DOI 10.17602/M2/M167726. DOI 10.17602/M2/M167727. DOI 10.17602/M2/M167728. DOI 10.17602/M2/M167729. DOI 10.17602/M2/M167730. DOI 10.17602/M2/M167731. DOI 10.17602/M2/M167734. DOI 10.17602/M2/M167740. DOI 10.17602/M2/M167743. DOI 10.17602/M2/M167744. DOI 10.17602/M2/M167747. DOI 10.17602/M2/M167759. DOI 10.17602/M2/M167760. DOI 10.17602/M2/M167761. DOI 10.17602/M2/M167762.
